# Metabolomics Reveals Dynamic Metabolic Changes Associated with Age in Early Childhood

**DOI:** 10.1371/journal.pone.0149823

**Published:** 2016-02-25

**Authors:** Chih-Yung Chiu, Kuo-Wei Yeh, Gigin Lin, Meng-Han Chiang, Shu-Chen Yang, Wei-Ju Chao, Tsung-Chieh Yao, Ming-Han Tsai, Man-Chin Hua, Sui-Ling Liao, Shen-Hao Lai, Mei-Ling Cheng, Jing-Long Huang

**Affiliations:** 1 Department of Pediatrics, Chang Gung Memorial Hospital at Keelung, and Chang Gung University, Taoyuan, Taiwan; 2 Community Medicine Research Centre, Chang Gung Memorial Hospital, Keelung, Taiwan; 3 Department of Pediatrics, Chang Gung Memorial Hospital at Linkou, and Chang Gung University, Taoyuan, Taiwan; 4 Department of Medical Imaging and Intervention, Chang Gung Memorial Hospital at Linkou, and Chang Gung University, Taoyuan, Taiwan; 5 Department of Nutrition Therapy, Chang Gung Memorial Hospital, Keelung, Taiwan; 6 Graduate Institute of Medical Biotechnology, Chang Gung University, Taoyuan, Taiwan; University of Arkansas for Medical Sciences, UNITED STATES

## Abstract

**Objectives:**

A detailed understanding of the metabolic processes governing rapid growth in early life is still lacking. The aim of this study was to investigate the age-related metabolic changes in healthy children throughout early childhood.

**Methods:**

Healthy children from a birth cohort were enrolled in this study from birth through 4 years of age. Urinary metabolites were assessed at 6 months, and 1, 2, 3, and 4 yr of age by using ^1^H-nuclear magnetic resonance (NMR) spectroscopy coupled with multivariate statistical analysis including principal components analysis (PCA) and partial least-squares discriminant analysis (PLS-DA). Metabolic pathway analysis was performed using the MetPA web tool.

**Results:**

A total of 105 urine samples from 30 healthy children were collected and analyzed. Metabolites contributing to the discrimination between age groups were identified by using supervised PLS-DA (Q^2^ = 0.60; R^2^ = 0.66). A significantly higher urinary trimethylamine *N*-oxide (TMAO) and betaine level was found in children aged 6 months. Urinary glycine and glutamine levels declined significantly after 6 months of age and there was a concomitant compensatory increase in urinary creatine and creatinine. Metabolic pathway analysis using MetPA revealed similar nitrogen metabolism associated energy production across all ages assessed. Pathways associated with amino acid metabolism were significantly different between infants aged 6 months and 1 year, whereas pathways associated with carbohydrate metabolism were significantly different between children at ages 2 and 3 years.

**Conclusions:**

Urine metabolomics ideally represents dynamic metabolic changes across age. Urinary metabolic profiles change significantly within the first year of life, which can potentially provide crucial information about infant nutrition and growth.

## Introduction

Metabolism refers to all biological processes and pathways in the body. Enzymes play a key role in many metabolic processes and functional changes. Consequently, genetic mutation of genes encoding enzymes can lead to problems in these pathways [1]. Growth in early life not only involves increasing the length and weight of a body, but is also accompanied by a complex remodeling of the immune system, endocrine system, and metabolism [2,3]. The incidence of certain diseases varies with age in children, including atopic diseases, hematological malignancies, autoimmune diseases, and diabetes. Understanding the complexity of age-related changes in metabolic profiles during early life is of utmost importance for the management of health and disease in childhood [4,5]. A detailed understanding of the metabolic processes during early childhood will likely provide much needed clinical insights.

Metabolic profiling provides a new opportunity to explore the global metabolic effects of many conditions on complex biological systems [6,7]. High resolution ^1^H-nuclear magnetic resonance (NMR) spectroscopy is widely used to quantitatively analyze metabolic profiles, due to its reliability and relatively straightforward sample preparation [8,9]. The metabolites in urine provide a fingerprint for each individual, containing significant information about age, sex, lifestyle, dietary intake, and disease history [10–12]. Metabolites that are correlated with age have been reported in the pediatric population, and include trimethylamine *N*-oxide (TMAO), citrate, creatine, glycine, succinate, acetone and creatinine [13,14]. However, metabolites definitely associated with the growth spurt during early childhood have not yet been elucidated.

The field of pediatric metabolomics is still being pioneered [15,16]. It is tremendously important to understand the dynamic metabolic changes that occur during infancy and childhood to further metabolomic research in pediatrics. The major aim of this study was to identify the determinants of urinary metabolic profiles in healthy children from a birth cohort, aged six months through to 4 years. The metabolite changes in urine associated with the sex of the child, breastfeeding patterns and age were assessed, and the likely metabolic pathway functioning was also examined.

## Methods

### Study population

A time-series study was designed to investigate the urinary metabolic profiles of healthy children from six months of age to 4 years. The urine samples were collected from healthy children that had been recruited as part of a birth cohort (the PATCH study) that was launched in 2007 to investigate the epidemiology and predictive factors of asthma and allergies in Taiwanese children. Detailed descriptions of subject recruitment and data collection have been reported previously [17,18]. Children without a personal history of asthma infections, or other atopic conditions were diagnosed in fifty-eight children for a 4-year follow-up. Thirty of the fifty-eight healthy children having urine samples taken over at least 3 time-points during the follow-up period were enrolled. This study was approved by the Institutional Review Board of Chang Gung Memorial Hospital (No. 102-1842C). Written informed consent was obtained from the parents or guardians of all study subjects.

### Definition of breastfeeding history

Detailed information on breastfeeding was obtained by well-trained investigators at 6 months of age. All children received supplemental food after 6 months of age. Infants who were fed with breast milk only, without additional food or drink, except water, were considered as the exclusive breastfeeding (EBF) set. Infants who were fed with formula only, without additional food or drink, except water, were defined as the formula feeding set. The set of partial breastfeeding (PBF) infants were those whose mother provided formula or other supplemental foods in addition to breast milk.

### Sample preparation

Spot urine samples collected in the morning at 6 months (n = 21), and 1 (n = 23), 2 (n = 20), 3 (n = 18) and 4 (n = 23) years of age were selected and examined. Urine samples were stored at -80 degree Celsius in aliquots until required. For each use, an aliquot was thawed, used and any remnants discarded after completion of the experiment. After thawing, to stabilize the pH value across samples prior to spectrum acquisition, 900 μL of urine was mixed with 100 μL of phosphate buffer (1.5 M KH_2_PO_4_, pH 7.4) in deuterium water which containing 0.04% 3-(trimethylsilyl)-propionic-2,2,3,3-d_4_ acid sodium salt (TSP) as an internal chemical shift reference standard, 2 mM NaN_3_ as an inhibitor of bacterial contamination. Each sample was vortexed for 20 s and subsequently centrifuged at 12000g for 30 min at 4°C. After centrifugation, a 650 μL aliquot of the supernatant was transferred to a standard 5 mm NMR tube for analysis.

### ^1^H–Nuclear Magnetic Resonance (NMR) spectroscopy

^1^H-NMR spectra were acquired at 300K on a Bruker Avance 600 MHz spectrometer (Bruker-Biospin GmbH, Karlsruhe, Germany) equipped with a 5 mm CPTCI ^1^H cryoprobe at Chang Gung Healthy Aging Research Center, Taiwan. For each spectrum, 64 scans were collected into 64K computer data points using a spectral width of 10,000 Hz (10 ppm) during the relaxation time of 4 s. All 1D spectra were applied for analysis before Fourier transformation with zero-filled to exponential line-broadenings of 0.3 Hz. The acquired ^1^H-NMR spectra were manually phased, baseline-corrected, and referenced to the chemical shift of TSP (δ 0.0 ppm) using TopSpin 3.2 software (Bruker BioSpin, Rheinstetten, Germany).

### NMR data processing and analysis

The raw ^1^H-NMR spectra were imported into AMIX version 3.9.12 (Bruker BioSpin, Rheinstetten, Germany) for spectral bucket, spectral region exclusion and spectral normalization. ^1^H-NMR spectra were aligned on the TSP peak and normalized on the spectral area for calculating the concentration of each metabolite in the spectral peaks of each metabolite. The ^1^H-NMR spectra were subdivided into integrated regions of 0.01 ppm corresponding to the region of δ 0–10 ppm. Regions containing residual water (δ 4.745–4.845 ppm) and urea (δ 5.465–6.195 ppm) were excluded from the data set to avoid spectral interference of residual water and urea. The normalized ^1^H-NMR bucket data were then uploaded to MetaboAnalyst 3.0 (http://www.metaboanalyst.ca) for partial least squares-discriminant analysis (PLS-DA), to identify metabolites contributing to the discrimination between age groups. The spectral variables were mean-centered and scaled to unit variance, and 10-fold internal cross-validation was performed to evaluate the quality of the resulting statistical models by considering the diagnostic measures R^2^ and Q^2^ [19], describing the endpoint variation captured in regression model, and the variation reproduced in cross-validation, respectively. The cross validation revealed a Q^2^ value of greater than 0.5 is usually considered to be a good classification model [20]. The ratio Q^2^/R^2^ is a measure of cross-validation reproducibility and Q^2^/R^2^ above 0.5 are considered indicative of relevant associations [21]. A permutation test can evaluate whether the specific classification of the individuals in the two designed groups is significantly better than any other random classification in two arbitrary groups [22]. Differences by a classifying variable are compared to a distribution of differences in randomly selected subsets of the data.

Metabolites were identified using Chenomx NMR Suite 7.5 professional software (Chenomx Inc., Edmonton AB, Canada). Hierarchical clustering was performed and heat maps were created based on the Pearson distance measure and the Ward clustering algorithm, displayed for metabolites selected by analysis of variance (ANOVA) using a significance level of *P* < 0.05, and post-hoc analysis of Fisher’s LSD. The fold changes in metabolites between age groups were performed by the non-parametric Mann-Whitney test using MetaboAnalyst web server. Pathway analysis of the metabolites was performed with Metabolomics Pathway Analysis (MetPA; http://metpa.metabolomics.ca/MetPA/faces/Home.jsp). The false discovery rate (FDR) was used as a way to limit the number of false positive results given the multiple comparison issues posed by many metabolites in this study.

## Results

### Population characteristics

A total of 105 urine samples from 30 healthy children, with no diagnosed diseases over a 4-year follow-up, were used for analysis. Population characteristics and the growth status are shown in Table 1. There were 17 boys and 13 girls with an average gestational age of 37.7 ± 1.8 weeks, and birth weight of 2.9 ± 0.5 kg. Breastfeeding history was carefully reviewed until 6 months of age, and results were stratified into three groups: exclusive breastfeeding (n = 13, 43%), partial breastfeeding (n = 13, 43%) and formula feeding (n = 4, 13%). There was a growth spurt with 2.7-fold increase in weight and 1.4-fold increase in height at 6 months of age as compared to at birth.

**Table 1:** Population characteristics and growth of 30 healthy children during a 4-year follow-up.

		Age				
Variable	Birth	6 Months	1 Years	2 Years	3 Years	4 Years
Urine samples (n)		21	23	20	18	23
Sex, male	17 (57%)					
Maternal age (yr)	32.4 ± 4.4					
Gestational age (wk)	37.7 ± 1.8					
Breastfeeding until 6 mo						
Exclusive	13 (43%)					
Partial	13 (43%)					
Formula	4 (13%)					
Weight, kg (percentile)	2.9 ± 0.5 (36.0 ± 26.9)	7.7 ± 1.0 (50.8 ± 35.1)	9.2 ± 1.1 (46.7 ± 28.1)	12.1 ± 1.7 (54.3 ± 30.9)	14.9 ± 1.9 (58.9 ± 30.0)	16.4 ± 2.6 (50.8 ± 31.4)
Height, cm (percentile)	48.2 ± 4.2 (56.1 ± 29.9)	67.1 ± 3.1 (53.3 ± 33.3)	74.9 ± 2.8 (50.1 ± 33.3)	86.9 ± 4.0 (49.1 ± 30.1)	95.4 ± 3.4 (40.6 ± 27.9)	102.2 ± 4.6 (45.1 ± 32.5)
BMI, kg/m^2^ (percentile)	12.8 ± 3.0 (33.7 ± 28.1)	17.2 ± 1.3 (49.1 ± 29.1)	16.3 ± 1.4 (44.5 ± 30.7)	15.9 ± 2.0 (52.1 ± 34.9)	16.3 ± 1.4 (68.8 ± 29.8)	15.6 ± 2.0 (50.7 ± 32.2)

yr, year; wk, week; mo, month; kg, kilograms; cm, centimeters; BMI, body mass index.

Data shown are mean ± SD or number (%) of subjects as appropriate. Percentile curves were calculated using the World Health Organization (WHO) charts.

### Identification of metabolite sets between sex, breastfeeding patterns and age groups

^1^H-NMR data of urine samples collected at different years of age were analyzed (See S1 Dataset). One thousand buckets varied across age groups, of which 247 buckets corresponded to 76 known metabolites (S1 Table). Unsupervised principal components analysis, followed by examination of the first three principal components, failed to separate groups clearly based on the sex of child, patterns of breastfeeding, or age groups. The PLS-DA parameters and permutation test used for distinguishing between the sexes, patterns of breastfeeding, and age groups are shown in Tables 2 and 3 respectively. Relevant associations (Q^2^/R^2^ > 0.5) were found between the sets with different patterns of breastfeeding, and between the different age groups. Metabolites identified in sets with different patterns of breastfeeding, and the different age groups, selected by using the cutoff of PLS-DA Variable Importance in Projection (VIP) score > 1.5 and a *P* value < 0.05 in the fold change of expression level, are shown in Tables 4 and 5 respectively. The metabolic profiles of urine changed significantly with breastfeeding patterns and age within the first year of life. However, a good classification model (Q^2^ > 0.5) with significant cross-validated value (*P* < 0.05 by permutation test) was only found between age groups, which were then studied further.

**Table 2:** PLS‐DA parameters and permutation test for distinguishing between gender and patterns of breastfeeding.

	Group Numbers	PLS‐DA parameters					Group Numbers	PLS‐DA parameters				
Age (yr)	(male–female)	Components^a^	Q^2^	R^2^	Q^2^/ R^2^	P_permutation_^b^	(EBF–PBF)	Components^a^	Q^2^	R^2^	Q^2^/ R^2^	P_permutation_^b^
0.5	13–8	1	-0.18	0.49	-0.37	0.19	9–10	1	0.34	0.66	0.52	0.38
1	16–7	1	0.09	0.64	0.14	0.45	8–12	1	0.17	0.67	0.25	0.14
2	14–6	1	-0.61	0.67	-0.79	0.73	5–11	1	-0.74	0.85	-0.87	0.99
3	11–7	1	-0.52	0.46	-1.13	0.81	4–11	1	-0.86	0.45	-1.91	0.71
4	13–10	1	-0.06	0.56	-0.11	0.47	8–11	1	-0.26	0.53	-0.49	0.80

PLS-DA, partial least squares-discriminant analysis; yr, year; Q^2^, predictive capability; R^2^, correlation coefficients; EBF, exclusive breastfeeding; PBF, Partial breastfeeding.

^a^The number of components based on Q^2^ indicates the best classifier of PLS-DA using a 10-fold cross-validation method.

^b^100 random permutations were performed.

**Table 3:** PLS‐DA parameters and permutation test for distinguishing between age groups.

		PLS‐DA parameters				
Age (yr)	Group Numbers	Components^a^	Q^2^	R^2^	Q^2^/R^2^	P_permutation_^b^
0.5–1	21–23	1	0.682	0.756	0.90	0.01
1–2	23–20	2	0.119	0.750	0.16	0.04
2–3	20–18	3	0.086	0.803	0.11	0.24
3–4	18–23	1	-0.243	0.448	-0.54	0.41

PLS-DA, partial least squares-discriminant analysis; yr, year; Q^2^, predictive capability; R^2^, correlation coefficients.

^a^The number of components based on Q^2^ indicates the best classifier of PLS-DA using a 10-fold cross-validation method.

^b^100 random permutations were performed.

**Table 4:** The VIP score and fold change of metabolites significantly differentially expressed between exclusive and partial breastfeeding at different years of age.

		Age 0.5				Age 1			Age 2			Age 3		
Metabolites	Chemical shift	VIP score		Fold change	*P*	VIP score	Fold change	*P*	VIP score	Fold change	*P*	VIP score	Fold change	*P*
Creatine	3.935(s)		6.86	0.55	**0.010**	6.97	0.76	**0.038**	1.96	1.08	0.827	3.90	0.88	0.679
Glycine	3.575–3.565(s)		4.66	1.28	**0.013**	5.16	1.30	**0.016**	5.42	0.79	0.090	3.01	0.88	0.254
Fucose	1.265–1.246(d)		3.65	1.49	**0.004**	2.31	1.16	**0.025**	-	1.01	0.827	-	0.96	0.953
Glutamine	2.465–2.425(m)		3.45	1.17	**0.022**	-	1.03	0.824	-	1.01	0.510	-	1.00	1.000
Hippuric acid	7.575–7.545(n)		3.41	0.49	**0.006**	3.32	0.61	**0.004**	4.24	1.52	0.320	5.31	0.58	0.859
Lysine	1.915–1.865(m)		2.74	0.87	**0.017**	-	0.99	1.000	2.27	0.92	0.221	-	0.99	1.000
*N*-Acetylglucosamine	2.055–2.045(m)		2.73	1.14	**0.028**	3.46	1.17	**0.025**	-	1.01	0.913	-	0.95	0.953
Methylmalonic acid	1.235–1.226(d)		2.45	1.30	**0.022**	-	1.08	0.295	-	0.93	0.510	1.98	0.89	0.310
Carnitine	3.235–3.225(s)		2.20	0.81	**0.028**	3.28	0.79	0.067	-	1.10	0.743	-	0.98	0.440
3-Methyl-2-oxovaleric acid	1.115–1.106(d)		2.01	0.65	**0.006**	1.84	0.76	**0.020**	-	1.11	0.510	2.35	1.41	0.099
3-Hydroxyisovaleric acid	2.365(s)		1.78	1.23	**0.006**	-	1.12	0.131	-	0.97	0.827	-	0.96	0.594
Acetic acid	1.925(s)		3.68	1.75	0.079	7.44	2.69	**0.020**	3.37	0.80	0.267	-	1.06	1.000
Formic acid	8.465–8.455(s)		1.50	1.36	0.356	2.96	1.63	**0.010**	2.38	0.69	0.145	-	0.91	0.768
Galactose	5.285–5.276(d)		-	0.86	0.497	2.36	0.50	**0.020**	1.63	1.59	0.090	1.81	1.69	0.371
Pantothenic acid	0.895(s)		-	0.96	0.549	1.59	0.80	**0.031**	-	1.14	0.267	-	1.15	0.254
Hypoxanthine	8.215–8.196(s)		-	0.77	0.113	-	1.03	0.552	2.15	1.50	**0.013**	-	0.89	0.594
2-Phenylpropionic acid	1.415(d)		-	0.80	**0.017**	-	0.84	0.503	-	1.26	0.267	2.60	1.54	**0.019**

VIP, Variable Importance in Projection; s, singlet; d, doublet; m, multiplet; n, nonet.

VIP scores were obtained from PLS-DA model and a VIP score < 1.5 was shown as “-”.

Fold change was calculated by dividing the value of metabolites in children receiving exclusive breastfeeding by partial breastfeeding.

All *P* values < 0.05, which is in bold, are significant.

**Table 5:** The VIP score and fold change of metabolites significantly differentially expressed between age groups.

		Age 0.5–1			Age 1–2			Age 2–3			Age 3–4		
Metabolites	Chemical shift	VIP score	Fold change	*P*	VIP score	Fold change	*P*	VIP score	Fold change	*P*	VIP score	Fold change	*P*
Trimethylamine *N*-oxide	3.275–3.265(s)	10.99	0.52	**<0.001**	4.71	0.87	0.142	1.78	1.08	0.393	4.01	1.17	**0.041**
Creatinine	3.045(s)	8.78	1.56	**<0.001**	-	1.04	0.352	-	0.81	0.176	-	1.14	0.928
Creatine	3.935(s)	7.48	2.04	**<0.001**	-	1.04	0.971	-	0.98	0.762	-	1.01	0.726
Betaine	3.905(s)	5.53	0.53	**<0.001**	1.51	0.95	0.055	-	0.97	0.158	1.83	1.22	0.328
Citric acid	2.575–2.515(d)	4.15	0.79	**<0.001**	-	0.99	0.782	2.92	0.93	0.426	-	0.97	0.687
*N*,*N*-Dimethylglycine	2.935–2.925(s)	3.45	0.57	**<0.001**	1.85	0.88	0.178	2.01	0.88	0.196	-	1.03	0.474
Galactose	3.515–3.485(dd)	2.90	0.80	**<0.001**	-	0.99	0.838	-	0.93	0.149	-	0.96	0.668
*N*-Phenylacetylglycine	7.375–7.355(m)	2.89	1.85	**<0.001**	2.18	1.15	0.462	2.96	1.20	0.217	2.45	0.86	0.256
Dimethylamine	2.715(s)	2.75	0.47	**<0.001**	-	0.93	0.352	-	1.03	0.099	-	1.01	0.886
Glycine	3.575–3.565(s)	2.57	0.82	**0.002**	2.21	1.09	0.240	2.10	0.90	0.346	1.57	1.06	0.668
Hippuric acid	7.575–7.545(n)	2.16	1.79	**0.008**	3.38	1.35	**0.034**	2.46	1.25	0.553	-	1.01	0.668
Glutamine	2.465–2.425(m)	1.81	0.88	**0.007**	1.55	0.96	0.365	1.91	0.93	0.228	2.07	1.07	0.316
*N*-Acetylglucosamine	2.055–2.045(m)	1.79	0.88	**0.008**	2.15	0.93	0.142	-	0.96	0.718	-	1.04	0.507
Lysine	1.915–1.865(m)	1.50	0.92	**0.027**	-	0.97	0.462	2.03	1.08	0.066	-	0.97	0.472
Carnitine	3.235–3.225(s)	-	1.09	0.167	3.39	0.84	**0.044**	-	1.05	0.675	3.42	1.26	0.107
Formic acid	8.465–8.455(s)	-	0.85	0.316	2.40	0.70	**0.028**	-	0.83	0.082	-	1.00	0.706
Lactose	4.705–4.685(d)	-	0.67	**<0.001**	1.74	0.77	**0.023**	3.66	3.23	**0.005**	2.80	0.47	0.948
3-Methylhistidine	7.015–7.005(s)	-	1.37	**0.042**	1.51	1.32	**0.046**	-	0.99	0.317	-	1.00	0.687
Acetic acid	1.925(s)	-	1.33	0.304	-	0.99	0.507	1.56	0.87	**0.015**	-	1.07	0.256

VIP, Variable Importance in Projection; s, singlet; d, doublet; dd, doublet of doublets; m, multiplet; n, nonet.

VIP scores were obtained from PLS-DA model and a VIP score < 1.5 was shown as “-”.

All *P* values < 0.05, which is in bold, are significant.

### Quantification of urinary metabolites across different years of age

**Fig 1:**
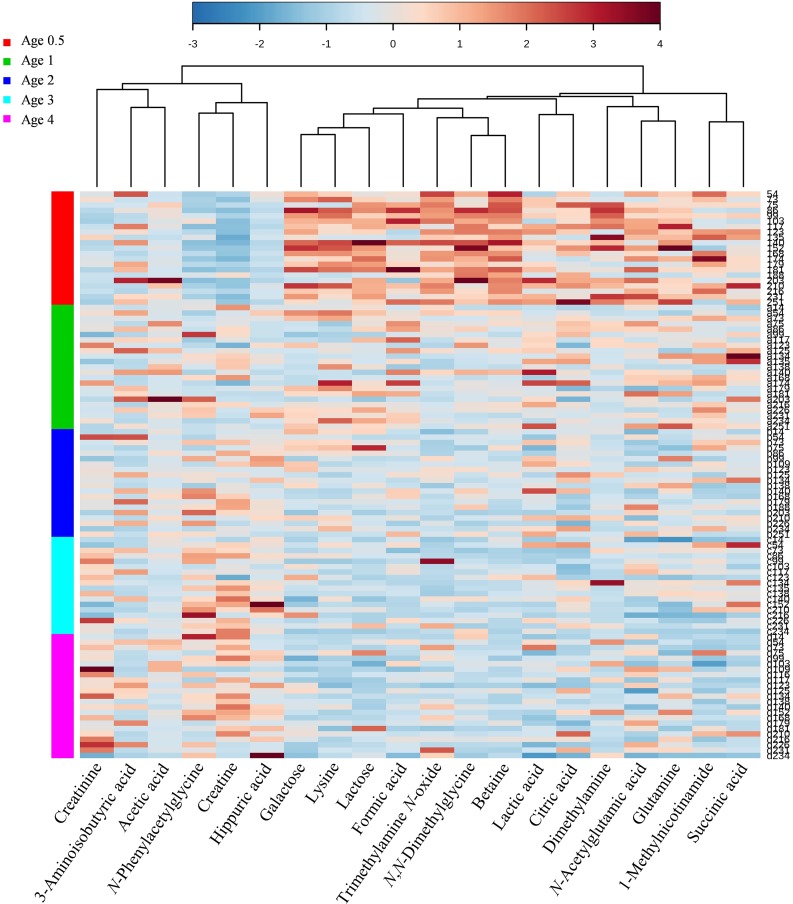
Heat map of 20 metabolites selected by the PLS-DA VIP score > 1.5 across 6 months to 4 years of age. Each row represents a urine sample and each column represents the expression profile of a metabolite across age groups. The changes of x-fold standard deviation from the overall mean concentration for different years of age are shown in a color-coded way. Blue color represents a decrease, and red color an increase. PLS-DA, partial least squares-discriminant analysis; VIP, Variable Importance in Projection.

**Fig 2:**
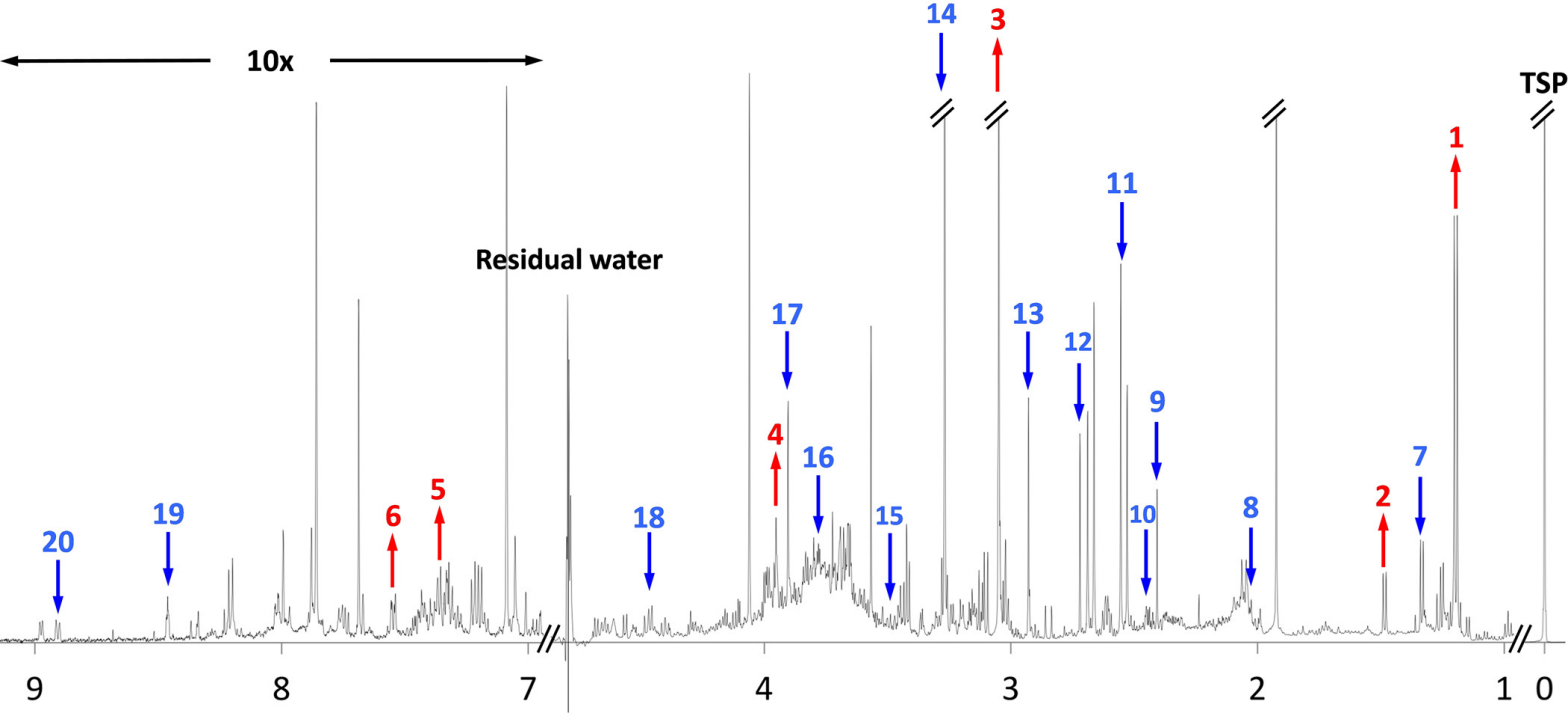
Representative 600 MHz ^1^H-NMR spectra of urine showing the metabolite signals of 20 age-related metabolites (δ1–9). With increasing age, red up arrow indicates a detected increase in metabolite concentration, whereas down blue arrow indicates a detected decrease in metabolite concentration. 1, 3-Aminoisobutyric acid; 2, acetic acid; 3, creatinine; 4, creatine; 5, *N*-phenylacetylglycine; 6, hippuric aicd; 7, lactic acid; 8, *N*-acetylglutamic acid; 9, succinic acid; 10, glutamine; 11, citric acid; 12, dimethylamine; 13, *N*,*N*-dimethylglycine; 14, Trimethylamine *N*-oxide; 15, galactose; 16, lysine; 17, betaine; 18, lactose; 19, formic acid; 20, 1-methylnicotinamide.

Metabolites distinguishing among age groups were identified by using supervised PLS-DA (Q^2^ = 0.60; R^2^ = 0.66). A total of 20 metabolites with VIP scores greater than 1.5 with a *P* value < 0.05 by ANOVA were identified across different years of age. Fig 1 shows a heat map of metabolites varied significantly across age groups by using Hierarchical Clustering. The fold changes from the overall mean concentration for different years of age are shown using color-codes. Fourteen metabolites decreased their concentration in urine with increasing age (galactose, lysine, lactose, formic acid, trimethylamine *N*-oxide (TMAO), *N*,*N*-dimethylglycine, betaine, lactic acid, citric acid, dimethylamine, *N*-acetylglutamic acid, glutamine, 1-methylnicotinamide and succinic acid). In contrast, six metabolites appeared to increase their concentration in urine with increasing age (creatinine, 3-aminoisobutyric acid, acetic acid, *N*-phenylacetylglycine, creatine and hippuric acid), as shown in Fig 2.

### Metabolic pathway and function analysis

Metabolites selected by filtering the dataset using the cutoff of PLS-DA VIP score > 1.5 between the different age groups were analyzed using MetPA, which is a free web-based tool that combines results from powerful pathway enrichment analysis with the topology analysis. Metabolites with concentrations that had altered were mapped onto likely relevant pathways, and were used to explain the metabolism. Metabolic pathway analysis with MetPA by filtering the dataset using a FDR-adjusted *P* value < 0.05 revealed that metabolites identified between age groups were all important for energy and were responsible for nitrogen metabolism (Table 6). Metabolic pathways associated with aminoacyl-tRNA biosynthesis were significantly different between the ages of 6 months and 1 year, and between the ages of 3 years and 4 years. In addition, the metabolism of the amino acids glycine, serine, and threonine was significantly different between the ages of 6 months and 1 year. In contrast, metabolism of galactose, a carbohydrate, was significantly different between the ages of 2 and 3 years.

**Table 6:** Metabolic pathway and function analysis between age groups.

Age	Pathway Name	Total	Hits	Metabolites	Raw *P*	FDR	Impact	Function
0.5–1	Glycine, serine and threonine metabolism	48	4	Glycine, Betaine, Creatine, *N*,*N*-Dimethylglycine	<0.001	0.017	0.246	Amino acid
	Aminoacyl-tRNA biosynthesis	75	4	Histidine, Glutamine, Glycine, Lysine	0.001	0.034	0.056	Genetic Information Processing; Translation
	Methane metabolism	34	3	Trimethylamine *N*-oxide, *N*,*N*-Dimethylglycine, Glycine	0.001	0.034	0.001	Energy
	Nitrogen metabolism	39	3	Histidine, Glutamine, Glycine	0.002	0.038	0.000	Energy
1–2	Nitrogen metabolism	39	5	Formic acid, Histidine, Taurine, Glutamine, Glycine	<0.001	0.002	0.000	Energy
2–3	Nitrogen metabolism	39	4	Histidine, Taurine, Glutamine, Glycine	<0.001	0.033	0.000	Energy
	Galactose metabolism	41	4	Galactose, Lactose, Galactitol, Glucose 6-phosphate	<0.001	0.033	0.346	Carbohydrate
3–4	Nitrogen metabolism	39	4	Histidine, Taurine, Glutamine, Glycine	<0.001	0.014	0.000	Energy
	Aminoacyl-tRNA biosynthesis	75	5	Histidine, Glutamine, Glycine, Lysine, Threonine	<0.001	0.014	0.056	Genetic Information Processing; Translation

Total is the total number of compounds in the pathway; the Hits is the actually matched number from the user uploaded data; the Raw *P* is the original *P* value calculated from the enrichment analysis; the false discovery rate (FDR) is the portion of false positives above the user-specified score threshold; the Impact is the pathway impact value calculated from pathway topology analysis.

## Discussion

Metabolomics involves the systematic study of endogenous small molecules that characterize the metabolic pathways of biological systems [23]. The first five years of a child’s life are a time of incredible growth and learning. It is believed that the age-dependent changes reflected in their metabolic profiles demonstrate the rapid growth occurring in early life. This study provides an overview of the dynamic metabolic changes from 6 months to 4 years of age, which potentially provide information on dietary patterns and rapid physical growth during early childhood.

As infants and children progress through a series of growth stages, the child’s nutrient needs correspond to these changes in growth rate. Parents should provide a diet with a wide variety of foods suited to the child’s age. In Taiwan, until 6 months of age, milk remains the primary food source. After this time, complementary or protein-rich solid foods are gradually added. Intake of carbohydrate-based staple foods such as rice, bread, and noodles mainly starts from the age of 1. In this study, the metabolic profiles associated with breastfeeding patterns appear to be significantly different at 6 months of age. Furthermore, amino acid and carbohydrate metabolisms changed significantly within in the first and second years respectively. These findings indicate that metabolomics can be used to analyze the dietary patterns characterized by different nutrition regimes in childhood.

In infancy, growth is rapid and affects different parts of the body at different rates [24]. An infant generally doubles birth weight by age 4 to 6 months and triples the birth weight within 1 year of birth, as was seen in this study [25]. In addition, dietary protein provides an important anabolic drive for the spurt of linear bone growth during infancy [26]. An increase in body mass increases the basal metabolic rate, or the amount of calories the body burns while at rest. In this study, urinary metabolites significantly changed within the first year. Metabolites resulting from nitrogen metabolism and energy production were identified throughout all of early childhood. These findings may support the idea that an infant needs more nutrition, in relation to size, height, and weight, than a preschool-age child needs.

Nitrogen metabolism and nitrogen compounds are important and essential components in the life-cycle of living organisms [27,28]. During childhood growth, the diet should provide adequate energy sources and assist nitrogen retention in the body as protein, resulting in weight gain commensurate with age. Muscle mass is the major reservoir of protein in human body, and changes in muscle mass can be detected in the urine as an altered nitrogen balance. Glycine is one of the non-essential amino acids, and is known to be required for the production of muscle tissue [29]. Glutamine is an important component of muscle protein, and helps repair and build muscle [30]. In this study, urinary glycine and glutamine levels declined significantly within in the first year, which may be interpreted, perhaps, by their use in increased growth of skeletal muscle tissue during infancy.

Creatinine is a chemical waste molecule of muscle metabolism and is excreted in urine at a relatively constant rate through glomerular filtration [31,32]. Creatinine is produced from creatine, a molecule of major importance for energy production in muscles. In this study, urinary creatine and creatinine levels appeared to increase with increasing age, which is consistent with the increase in muscle metabolism needed for a broad range of physical activities following early infancy. Generally, urinary creatinine is often used to adjust for urine analyte concentrations because of its relative stability within an individual. However, it must be emphasized that a strict assessment of the normal reference values of urinary creatinine based on the subjects’ age is considered a crucial step to investigate metabolic diseases in children using urine samples.

Trimethylamine *N*-oxide (TMAO), produced by bacteria in the intestine, is a product of the oxidation of trimethylamine. The blood level of TMAO increases after consuming foods containing carnitine in red meat [33] or after consuming lecithin in soy, eggs and milk [34,35]. Lecithin is the major dietary source of choline. Choline and its metabolites, such as betaine, can serve as a source of methyl groups required for numerous cellular functions such as DNA methylation, phosphatidylcholine biosynthesis, and protein biosynthesis [36]. In this study, a significantly higher urinary TMAO and betaine level was found in children at age 6 months, at which age milk is the primary source of nutrition, compared to all other age groups. Clinically, TMAO has been reported to be associated with cardiovascular and chronic kidney diseases [34,37], indicating that metabolomics not only detects likely dietary patterns, but can also point to early metabolic alterations that can be targeted in preventive interventions to prevent diet-related conditions.

Metabolomics has been used to study human diseases extensively, and has resulted in significant advances in the understanding of their pathophysiology [38]. In this study, metabolism of aminoacyl-tRNA biosynthesis and nitrogen metabolism appeared to be particularly active during early childhood. Aminoacyl-tRNAs, substrates for translation, are pivotal in determining how the genetic code is interpreted as amino acids, and mutations in aminoacyl-tRNA synthetases have been reported to be associated with mitochondrial metabolic diseases and congenital heart diseases [39–41]. Our findings suggest that further studies using this approach may be useful to investigate unexplained diseases that occur early in childhood.

Limitations of this study include the small sample size with limited statistical power for subanalyses, and the low sensitivity of ^1^H-NMR-based metabolomic analysis relative to mass spectrometry-based methods. Urinary creatinine concentrations are known to be different among age groups; and so urinary creatinine was not used to adjust the concentrations of urine analyte in this study. Although the time of day, in the morning, at which urine samples were collected, produced only a small variation in dilution effects of samples, there is still a limitation on the interpretation of our findings, because no normalization of metabolite levels against urinary creatinine was performed in the same age group. However, a significant strength of the present study lies in its longitudinal design, allowing long-term, sequential measurements of urinary metabolites at very close intervals.

In conclusion, to our knowledge, this is the first study to identify a group of urinary metabolites at very close intervals during early childhood using a ^1^H NMR-based metabolomic approach. Metabolomics can reveal dynamic metabolic changes across age, emphasizing the importance of using age matched samples for metabolic studies in infants and young children. The metabolic profiles of urine appear to change significantly within the first year of age, during which time a child’s diet changes from milk to more solid foods, and a physical growth spurt occurs. Age-related metabolites and their metabolic pathways could potentially provide crucial information about infant nutrition and growth. However, further studies with a larger sample size, especially for subgroup analysis, are needed to investigate any associations more comprehensively.

## Supporting Information

S1 Dataset Raw normalized ^1^H-NMR data of urine samples at 6 months, and 1, 2, 3, and 4 yr of age. (XLSX)

S1 Table ^1^H-NMR assignment results of the identified metabolites, chemical shifts and VIP scores in urine samples. (DOCX)
