# Supplementary material for: Metabolomics Reveals Dynamic Metabolic Changes Associated with Age in Early Childhood
Source: PLoS One. 2016 Feb 25;11(2):e0149823. doi: 10.1371/journal.pone.0149823 (PMC4767415; doi:10.1371/journal.pone.0149823)
Supplement: S1 Table — (DOCX) [file pone.0149823.s002.docx]

**Table S1 ^1^H-NMR assignment results of the identified metabolites, chemical shifts and VIP scores in urine samples.**

| Metabolites | Chemical shift  ppm (multiplicity) | VIP score |
| --- | --- | --- |
| Creatinine | 3.045(s) | 9.63 |
| Trimethylamine *N*-oxide | 3.275-3.265(s) | 6.72 |
| Creatine | 3.935(s) | 5.93 |
| Betaine | 3.905(s) | 5.23 |
| Citric acid | 2.575-2.515(d) | 4.76 |
| *N*-Phenylacetylglycine | 7.375-7.355(m) | 3.49 |
| *N*,*N*-Dimethylglycine | 2.935-2.925(s) | 3.47 |
| Hippuric acid | 7.575-7.545(n) | 3.38 |
| Galactose | 3.515-3.485(dd) | 3.16 |
| Succinic acid | 2.415-2.405(s) | 2.43 |
| Ethanol | 3.675(q) | 2.38 |
| Lactic acid | 1.345-1.315(d) | 2.29 |
| Glutamine | 2.465-2.425(m) | 2.17 |
| *N*-Acetylglucosamine | 2.055-2.045(m) | 2.14 |
| Galactitol | 3.705-3.685(m) | 1.97 |
| Dimethylamine | 2.715(s) | 1.96 |
| 3-Aminoisobutyric acid | 1.205-1.185(d) | 1.94 |
| Formic acid | 8.465-8.455(s) | 1.92 |
| Acetic acid | 1.925(s) | 1.92 |
| Glycine | 3.575-3.565(s) | 1.86 |
| Aminoadipic acid | 2.275(t) | 1.53 |
| Alanine | 1.495-1.465(d) | 1.48 |
| 3-Hydroxyisovaleric acid | 2.365(s) | 1.36 |
| Malonic acid | 3.125(s) | 1.35 |
| Taurine | 3.445-3.415(t) | 1.34 |
| Guanidoacetic acid | 3.805-3.795 (s) | 1.32 |
| Histidine | 7.085(d) | 1.23 |
| Lactose | 4.705-4.685(d) | 1.21 |
| 2-Aminobutyric acid | 0.985(t) | 1.20 |
| 1-Methylnicotinamide | 8.985-8.965(d) | 1.19 |
| Creatine phosphat | 3.955(s) | 1.16 |
| Pantothenic acid | 0.935(s) | 1.12 |
| *N*-Acetyltyrosine | 6.865-6.875(d) | 1.11 |
| Fucose | 1.265-1.246(d) | 1.10 |
| Pyruvic acid | 2.375 (s) | 1.05 |
| Propylene glycol | 1.155-1.135(d) | 1.05 |
| Methylmalonic acid | 1.235-1.225(d) | 1.04 |
| *N*-Methylhydantoin | 4.095(s) | 1.04 |
| 3-Indoxylsulfate | 7.715-7.695(d) | 1.03 |
| 2-Hydroxyisobutyric acid | 1.365-1.355(s) | 0.97 |
| 3-Methylhistidine | 7.015-7.005(s) | 0.97 |
| 3-Methyl-2-oxovaleric acid | 1.115-1.106(d) | 0.78 |
| *N*-Acetyltyrosine | 7.175(d) | 0.77 |
| Isobutyric acid | 1.085-1.065(d) | 0.77 |
| Carnitine | 3.235-3.225(s) | 0.77 |
| *N*-Nitrosodimethylamine | 3.175(s) | 0.76 |
| Aspartic acid | 2.805(dd) | 0.75 |
| Arabinose | 4.515(d) | 0.75 |
| Glucose | 4.675(d) | 0.70 |
| 3-Chlorotyrosine | 6.995(dd) | 0.68 |
| 1-Methylhistidine | 3.735(s) | 0.68 |
| Lysine | 1.915-1.865(m) | 0.67 |
| dTTP | 7.685(d) | 0.65 |
| Isoleucine | 1.015-1.005(d) | 0.61 |
| Glucuronic acid | 5.255(d) | 0.61 |
| 2-Phenylpropionic acid | 1.415(d) | 0.61 |
| Cis-Aconitic acid | 3.115(d) | 0.60 |
| Leucine | 0.955(t) | 0.59 |
| Allantoin | 5.395 (s) | 0.57 |
| *N*,*N*-Dimethylformamide | 2.855(d) | 0.56 |
| Hypoxanthine | 8.215-8.196(s) | 0.52 |
| Maltose | 5.405(d) | 0.51 |
| Gluconic acid | 4.145(d) | 0.46 |
| Choline | 3.185(s) | 0.42 |
| 3-Phenylpropionic acid | 2.495(t) | 0.42 |
| Homogentisic acid | 6.715-6.705(m) | 0.39 |
| Xanthine | 7.935(s) | 0.34 |
| 4-Pyridoxic acid | 7.865(s) | 0.29 |
| Valine | 1.055-1.035(d) | 0.28 |
| Fumaric acid | 6.525(s) | 0.27 |
| Dimethyl sulfone | 3.155 (s) | 0.19 |
| 3-Hydroxymandelic acid | 4.935(s) | 0.15 |
| Imidazole | 7.335(s) | 0.14 |
| Niacinamide | 8.675-8.655(dd) | 0.10 |
| NAD+ | 9.365(s) | 0.09 |
| NADP+ | 8.815(d) | 0.07 |

VIP, Variable Importance in Projection; s, singlet; d, doublet; m, multiplet; n, nonet; dd, doublet of doublets; q, quartet; t, triplet.
